# Regional differences in the prevalence of diabetic retinopathy: a multi center study in Brazil

**DOI:** 10.1186/s13098-018-0319-4

**Published:** 2018-03-14

**Authors:** Karla Rezende Guerra Drummond, Fernando Korn Malerbi, Paulo Henrique Morales, Tessa Cerqueira Lemos Mattos, André Araújo Pinheiro, Felipe Mallmann, Ricardo Vessoni Perez, Franz Schubert Lopes Leal, Laura Gomes Nunes de Melo, Marília Brito Gomes, The Brazilian Type 1 Diabetes Study Group, Carlos Antonio Negrato, Roberta Cobas, Lucianne Righeti Monteiro Tannus, Melanie Rodacki, Lenita Zajdenverg, Joana Rodrigues Dantas, Maria Lúia Cardillo Corrêa-Giannella, Sharon Nina Admoni, Daniele Pereira dos Santos, Maria de Fatima Guedes, Sergio Atala Dib, Celso Ferreira de Camargo Sallum Filho, Elisabeth João Pavin, Caroline Takano, Rosângela Roginski Rea, Nicole Balster Romanzini, Mirela Azevedo, Luis Henrique Canani, Hermelinda Cordeiro Pedrosa, Monica Tolentino, Cejana Hamu Aguiar, Reine Marie Chaves Fonseca, Ludmila Chaves Fonseca, Raffaele Kasprowicz, Adriana Costa e Forti, Angela Delmira Nunes Mendes, Renan Montenegro Junior, Virgínia Oliveira Fernandes, João Soares Felício, Flavia Marques Santos

**Affiliations:** 1 Universidade do Estado do Rio de Janeiro, Boulevard 28 de Setembro, 77, Vila Isabel, Rio de Janeiro, RJ Zip Code: 20551-030 Brazil; 2 Universidade Federal de São Paulo, São Paulo, Brazil; 3 Centro de Endocrinologia e Diabetes do Estado da Bahia, Salvador, Brazil; 4 Hospital Regional de Taguatinga, Brasília, Brazil; 5 Hospital de Clinicas de Porto Alegre, Porto Alegre, Rio Grande do Sul Brazil; 6 Universidade de São Paulo, São Paulo, Brazil; 7 Universidade de Campinas, São Paulo, Brazil

**Keywords:** Diabetic retinopathy, Diabetic macular edema, Risk factors

## Abstract

**Background:**

Diabetic retinopathy has a significant impact in every healthcare system. Despite that fact, there are few accurate estimates in the prevalence of DR in Brazil’s different geographic regions, particularly proliferative DR and diabetic macular edema. This study aims to determine the prevalence of diabetic retinopathy in Brazil’s five continental regions and its determinant factors.

**Methods:**

This multi center, cross-sectional, observational study, conducted between August 2011 and December 2014, included patients with type 1 diabetes from the 5 Brazilian geographic regions (South, Southeast, North, Northeast and Midwest). During a clinical visit, a structured questionnaire was applied, blood sampling was collected and each patient underwent mydriatic binocular indirect ophthalmoscopy evaluation.

**Results:**

Data was obtained from 1644 patients, aged 30.2 ± 12 years (56.1% female, 54.4% Caucasian), with a diabetes duration of 15.5 ± 9.3 years. The prevalence of diabetic retinopathy was 242 (36.1%) in the Southeast, 102 (42.9%) in the South, 183 (29.9%) in the North and Northeast and 54 (41.7%) in the Midwest. Multinomial regression showed no difference in the prevalence of non-proliferative diabetic retinopathy in each geographic region, although, prevalence of proliferative diabetic retinopathy (p = 0.022), and diabetic macular edema (p = 0.003) was higher in the Midwest. Stepwise analyses reviled duration of diabetes, level of HbA1c and hypertension as independent variables.

**Conclusions:**

The prevalence of non proliferative diabetic retinopathy in patients with type 1 diabetes was no different between each geographic region of Brazil. The Midwest presented higher prevalence of proliferative diabetic retinopathy and diabetic macular edema. Duration of DM and glycemic control is of central importance to all. Hypertension is another fundamental factor to every region, at special in the South and Southeast. Glycemic control and patients in social and economic vulnerability deserves special attention in the North and Northeast of Brazil.

## Background

Diabetic retinopathy (DR) has a significant impact in the health care system. It is the leading cause of blindness among working-aged adults worldwide [1]. From 1990 to 2010, DR ranked as the fifth most common cause of preventable blindness and fifth most common cause of moderate to severe visual impairment [2, 3]. As a major public health problem causes productivity losses due to early retirement, suffering and diminishing quality of life, for millions of people. Vision disability is one of the top 10 disabilities among adults [4]. In Brazil, early retirement in patients due to diabetes-related chronic complications may result in more than 15 years of workforce loss per retired patient [5].

The increasing incidence of type 1 diabetes is a challenge for health care systems in many countries. Type 1 diabetes (T1D) is increasing by around 3% every year, particularly among children. Around 86,000 children develop type 1 diabetes each year and Brazil ranks as the third country in the world in number of children with T1D [6].

Despite the significance of this problem, [7], there are few accurate estimates of DR prevalence in Brazil, and even less in it’s different geographic regions, particularly severe vision-threatening stages of the disease, including proliferative DR (PDR) and diabetic macular edema (DME).

Brazil with its continental territory is the fifth largest country in the world, and the largest country of South America. Has an estimated population of 204.4 million people with 85.43% living in urban areas [8]. Brazil has diverse social, economic and educational realities as well as unequal access to public health care and education system. Generating a broader and more precise estimate of the prevalence of DR in each of the five regions of Brazil and its relationship with major modifiable risk factors is crucial for guiding specific public health education and optimal clinical management of diabetes. We therefore conducted a multi center study in Brazil to determine the prevalence of DR and its sight-threatening end points (PDR and DME) throughout its five continental regions and the determinants factors for each region.

## Methods

### Patients

This multi center, cross-sectional, observational study, conducted between August 2011 and December 2014, included patients with T1D from 14 public secondary (ambulatory out-patient clinics) and tertiary care-level clinics (ambulatory outpatient clinics in university hospitals) located in 10 different cities of the 5 Brazilian geographic regions (South, Southeast, North, Northeast and Midwest). Inclusion criterion was T1D according to the American Diabetes Association (ADA) [9]. Diagnosis was based on the typical clinical presentation, including variable degrees of weight loss, polyuria, polydipsia and polyphagia, and the need for continuous insulin use since the diagnosis of T1D. Antibody dosage was not done because of it’s limited availability in our public healthcare system. Exclusion criteria were pregnancy, lactation, recent history of acute infection or diabetic ketoacidosis 3 months preceding evaluation. All patients received healthcare from the Brazilian National Health Care System (Sistema Único de Saúde, SUS), that guarantees free healthcare for every Brazilian citizen. All eligible participating centers had a diabetes clinic with at least one endocrinologist which provided data from a minimum of 50 outpatients with diagnosis of T1D who regularly attended the clinic.

The local ethics committee approved the study protocol (CAAE: 0214.0.228.000-10) and written informed consent was obtained from all patients or their legal representative.

### Data collection

During a clinical visit, a structured questionnaire applied by trained physician accessed demographic data, clinical history, educational and economic status for each patient enrolled. The following variables were assessed during the interview: age, age at diagnosis, duration of diabetes, ethnicity, height (m), weight (kg), smoking status, self reported level of physical activity, level of education and economic status, previews diagnose and treatment of ophthalmological pathologies. Blood sampling was collected for determination of HbA1c levels (by high-performance liquid chromatography—HPLC, Bio-Rad Laboratories, Hercules, California, USA). Body mass index (BMI) was determined by dividing an individual’s weight (kg) by the square of their height (m^2^). Economic status was defined according to the Brazilian Economic Classification Criteria [10]. The following economic status categories were considered for this analysis: high, middle, low, and very low. All patients were referred for fundoscopy evaluation. To standardize procedure and evaluation, retina specialist from each center were assembled at one University center before the beginning of the study.

Each patient had both eyes examined and underwent mydriatic binocular indirect ophthalmoscopy. Mydriasis was obtained with 1% tropicamide drops. Binocular indirect ophthalmoscopy was performed with an Eyetec Ophthalmoscope (Eyetec, São Carlos-SP, Brazil) and a 20-diopter lens (Volk Optical, Mentor, OH, USA) by an experienced retinal specialist in each center. Each eye was classified for DR and DME according to the American Academy of Ophthalmology guidelines [11]. In brief, non-proliferative DR (NPDR) was graded into mild, when micro aneurysms or dot hemorrhages was found, moderate when more than mild and less then severe, and severe if hemorrhages (> 20) in four quadrants or venous beadings in two quadrants or intraretinal microvascular abnormalities in one quadrant was found. PDR was defined as disc neovascularization or elsewhere, vitreous hemorrhages or tractional retinal detachment. For patient classification, DR severity in the worst eye was considered.

### Sample calculation

The sample size of the present study was based on the Brazilian Multi center Type 1 Diabetes Study, described elsewhere [12]. The number of patients needed to be enrolled in each region was calculated based on the estimated prevalence of T1D and DR in Brazil [13] combined with the overall population density of each geographic region reported in the 2000 Brazilian Institute of Geography and Statistics Census (IBGE) (38.8, 2.6, 29.0, 23.0 and 6.6% in the Southeast, North, Northeast, South and Midwest regions, respectively) [14]. Because of the small number of patients needed to be enrolled at the North of Brazil, for statistical analysis, data from the North and Northeast regions were combined.

### Statistics

Statistical analyses were performed with SPSS 19.0 for Windows (SPSS Inc., Chicago, IL, USA). Data are presented as mean and standard deviation for continuous variable. Categorical variables are presented as absolute and relative frequency.

Comparisons between categorical variables were performed using exact Fisher test, where association was present Standard Residual was used to identify excess. Standard Residual (S.Res.) has normal distribution and is considered significant when absolute values are greater than 1.96. Positive values are considered excess, there are more cases than expected. Kruskal–Wallis (more than two groups) or Wilcoxon Test (two groups) were used for comparison between quantitative variables. When significant at the Kruskal–Wallis test multiple analyses were performed using the Wilcoxon test and Bonferroni correction. Linear model was used for further analyses with dependent variable being retinopathy classification group and independent variables the geographic regions and all of those with statistical significance or clinical relevance according to literature: age, sex, ethnicity, level of education, economic status, duration of diabetes, HbA1c, BMI, level of exercise, smoking, hypertension, hypercholesterolemia and level of care. Stepwise multiple regression analysis was further used for reduced model with the most important variables related with the disease. Level of significance was considered 5%.

## Results

### Overview of demographic and clinical data

Our study enrolled 1760 patients distributed in all five regions of Brazil, those of which 1644 underwent fundoscopy evaluation. Clinical and demographic data from the study population are shown in Table 1. Overall, 987 (56.1%) were females, with a duration of diabetes of 15.5 ± 9.3 years. Mean age was 30.2 ± 12 years and 958 (54.4%) were Caucasian. The tertiary health care accounted for 63.0% of the patients enrolled. Level of education was measured as years of study, which the mean was 12.2 ± 3.8 years. About half of the patients 910 (51.7%) were physically active and 227 (13.8%) reported smoking. Mean HbA1c was 9.0 ± 2.1%. Dyslipidemia was present in 21.3% of the patients and hypertension in 17.7%.

**Table 1:** Clinic and demographic data of the study population

Variables	
Age, years	30.2 ± 11
Gender, F (%)	987 (56.1%)
Duration of diabetes, years	15.5 ± 9.3
Ethnicity, n (%)	
Caucasian	958 (54.4%)
Non-Caucasian^a^	802 (45.6%)
Level of education, years	12.2 ± 3.8
Economic status	
High	53 (3%)
Medium	801 (45.5%)
Low	849 (48.2%)
Very low	57 (3.2%)
Geographic region (%)	
Southeast	829 (47.1%)
South	233 (13.2%)
North and Northeast	490 (27.8%)
Midwest	208 (11.8%)
Level of care, n (%)	
Secondary	651 (37%)
Tertiary	1109 (63%)
Physical activity	
Yes	910 (51.7%)
No	848 (48.2%)
Smoking	
Yes	227 (13.8%)
No	1414 (86.2%)

Data are presented as the means (SD) and n (%)

*F* female

^a^African-Brazilians, Mulattos, Asians, Native Indian

### Overview of ophthalmological data

Considering each geographic region, Table 2, the prevalence of DR was 242 (36.1%) in the Southeast, 102 (42.9%) in the South, 183 (29.9%) in the North and Northeast and 53 (41.7%) in the Midwest. In the North and Northeast of Brazil, prevalence of DR was significantly lower, p = 0.015. Prevalence of mild, moderate and severe NPDR was statistically similar in each geographic region of Brazil, varying from: 129 (19.2%) in the Southeast to 87 (14.2%) in the North and Northeast, 44 (6.6%) in the Southeast to 7 (5.5%) in the Midwest and 5 (0.8%) in the North and Northeast to 0 (0.0%) in the Midwest, respectively. The South had greater prevalence of PDR, [36 (15.1%) vs 17 (13.4%) in the Midwest, 64 (9.5%) in the southeast and 52 (8.5%) in the North and Northeast, p = 0.015]. In the matter of DME, prevalence in the Midwest region was higher [9 (7.1%) vs 17 (2.5%) in the Southeast, 3 (1.3%) in the South and 10 (1.6%) in the North and Northeast, p = 0.007].

**Table 2:** Clinic, demographic and laboratory data according to geographic regions

Variables	Southeast	South	North and Northeast	Midwest	p value
Age, years	31.0 ± 12.3	34.4 ± 12.8*	26.8 ± 9.8**	29.0 ± 11.7	0.000
Duration of diabetes, years	17.0 ± 9.7***	17.6 ± 9.9***	12.6 ± 7.7	14.1 ± 8.7	0.000
Level of HbA1c (%)	8.8 ± 2.0^+^	9.1 ± 2.1	9.3 ± 2.3	9.2 ± 2.1	0.003
Level of education, years	12.0 ± 3.5	12.0 ± 4.0	12.2 ± 4.0	13.2 ± 4.3^++^	0.004
BMI	24.5 ± 4.4	24.6 ± 3.8	23.5 ± 3.6**	23.9 ± 4.4	0.000
Physical activity, n (%)					
Yes	163 (38.1%)	60 (14%)	179 (41.8%)^#^	26 (6.1%)	0.039
No	577 (43.4%)	193 (14.5%)	457 (34.3%)	104 (7.8%)	
Smoking, n (%)					
Yes	97 (39.4%)	66 (26.8%)^##^	71 (28.9%)	12 (4.9%)	0.000
No	644 (42.5%)	187 (12.4%)	565 (37.3%)^a^	118 (7.8%)	
Hypertension					
Yes	137 (18.5%)	68 (26.9%)^c^	78 (12.3%)	23 (17.6%)	0.000
No	604 (81.5%)	185 (73.1%)	556 (87.7%)^c^	108 (82.4%)	
Hypercholesterolemia					
Yes	182 (24.7%)^e^	60 (23.7%)	100 (15.8%)	24 (18.5%)	0.001
No	556 (75.3%)	193 (76.3%)	533 (84.2%)^f^	106 (81.5%)	
Economic status, n (%)					
High	14 (1.9%)	7 (2.8%)	23 (3.6%)	8 (6.2%)^###^	0.000
Medium	380 (51.3%)^b^	142 (56.1%)^c^	188 (29.6%)	63 (48.5%)	
Low	335 (45.2%)	100 (39.5%)	375 (59.1%)^d^	58 (44.6%)	
Very low	12 (1.6%)	4 (1.6%)	49 (7.7%)^d^	1 (0.8%)	
Retinopathy stage					
DR absent	429 (63.9%)	136 (57.1%)	430 (70.1%)^e^	74 (58.3%)	0.015
Mild NPDR	129 (19.2%)	45 (18.9%)	87 (14.2%)	29 (22.8%)	
Moderate NPDR	44 (6.6%)	20 (8.4%)	39 (6.4%)	7 (5.5%)	
Sever NPDR	5 (0.7%)	1 (0.4%)	5 (0.8%)	0 (0.0%)	
Proliferative DR	64 (9.5%)	36 (15.1%)a	52 (8.5%)	17 (13.4%)	
DME	17 (2.5%)	3 (1.3%)	10 (1.6%)	9 (7.1%)^f^	0.007

Data are presented as the mean (SD) and N (%)

*BMI* body mass index, *DR* diabetic retinopathy, *NPDR* non-proliferative diabetic retinopathy, *DME* diabetic macular edema

* p < 0.001 vs every other region. ** p < 0.000 vs Southeast and South. *** p < 0.000 vs North and Northeast and Midwest. ^+^p = 0.003 vs North and Northeast. ^++^p < 0.049 vs Southeast and North and Northeast

^#^Standard Residual 2.8 ^##^ Standard Residual 5.6 ^###^ Standard Residual 2.3

^a^Standard Residual 2.6, ^b^ Standard Residual 5.3, ^c^ Standard Residual 4.2, ^d^ Standard Residual > 6.1, ^e^ Standard Residual 3.4, ^f^ Standard Residual 3.9

When questioned on ophthalmological pathologies and treatments, 109 (6.2%) referred cataract and 59 (3.4%) glaucoma. Prevalence of cataract and glaucoma was no different in each region of Brazil.

### Analysis of variables related to DR by geographic regions

#### Regions overview

Analysis of variables related to DR by geographic regions are shown in Tables 2, 3.

**Table 3:** Clinic, demographic and laboratory data by diabetic retinopathy classification according to geographic region

Variables	DR absent	Mild NPDR	Moderate NPDR	Sever NPDR	Proliferative DR	*p* value
Duration of diabetes, years Mean (SD)						
Southeast	14.2 ± 8.6*	19.0 ± 9.6**	22.3 ± 8.7	22.8 ± 7.8	25.7 ± 9.5	0.000
South	14.1 ± 9.3^##^	20.5 ± 9.5	19.8 ± 6.1	19.0 ± 0.0	25.7 ± 8.5	
North and Northeast	10.7 ± 6.8^+^	15.9 ± 7.3	15.5 ± 9.0**	15.3 ± 8.7	20.0 ± 7.9	
Midwest	10.0 ± 6.6*	17.3 ± 7.1**	22.0 ± 6.8	–	23.6 ± 9.2	
Level of HbA1c (%) Mean (SD)						
Southeast	8.7 ± 2.0***	9.3 ± 2.2	9.5 ± 2.6	8.4 ± 1.1	8.6 ± 1.4	0.012
South	8.8 ± 1.9	9.4 ± 2.1	10.0 ± 3.5	10.4 ± 0.0	8.9 ± 1.6	0.388
North and Northeast	9.0 ± 2.1^###^	9.1 ± 2.3^###^	11.1 ± 2.5	10.9 ± 3.6	9.5 ± 2.5	0.002
Midwest	9.1 ± 2.2	9.0 ± 2.1	9.5 ± 1.5	–	8.9 ± 1.8	0.698
Level of education, years Mean (SD)						
Southeast	12.1 ± 3.3	11.8 ± 3.9	12.1 ± 3.8	11.5 ± 1.9	11.9 ± 3.8	0.850
South	12.3 ± 3.6	12.3 ± 4.2	10.5 ± 3.8	13.0 ± 0.0	11.7 ± 5.1	0.307
North and Northeast	12.7 ± 3.9^###^	11.7 ± 4.1	10.0 ± 3.7	13.3 ± 4.6	11.2 ± 4.0	0.003
Midwest	13.1 ± 3.5	13.7 ± 5.3	12.3 ± 6.7	–	13.6 ± 4.7	0.736
BMI Mean (SD)						
Southeast	24.0 ± 3.9^#^	25.1 ± 4.9	26.3 ± 4.9	23.0 ± 6.3	26.3 ± 6.1	0.002
South	23.9 ± 4.0^++^	26.0 ± 2.8	24.5 ± 4.2	24.9 ± 0.0	25.1 ± 3.7	0.006
North and Northeast	23.3 ± 3.4	24.4 ± 4.2	24.9 ± 4.1	22.8 ± 1.3	23.0 ± 3.8	0.075
Midwest	23.1 ± 4.2**	24.7 ± 5.1	24.4 ± 3.4	–	25.8 ± 4.5	0.010
Level of care, n (%)						
Southeast						
Secondary	77 (70.6%)	18 (16.5%)	12 (11.0%)^a^	2 (1.8%)	0 (0.0%)	0.000
Tertiary	352 (62.5%)	111 (19.7%)	32 (5.7%)	4 (0.7%)	64 (11.4%)^d^	
South						
Secondary	0 (0.0%)	0 (0.0%)	0 (0.0%)	0 (0.0%)	0 (0.0%)	
Tertiary	136 (57.1%)	45 (18.9%)	20 (8.4%)	1 (0.4%)	36 (15.1%)	
North and Northeast						0.000
Secondary	254 (63.0%)	69 (17.1%)^c^	29 (7.2%)	3 (0.7%)	48 (11.9%)^f^	
Tertiary	175 (83.3%)^e^	18 (8.6%)	10 (4.8%)	3 (1.4%)	4 (1.9%)	
Midwest						
Secondary	74 (58.3%)	29 (22.8%)	7 (5.5%)	–	17 (13.4%)	
Tertiary	0 (0.0%)	0 (0.0%)	0 (0.0%)	–	0 (0.0%)	
Physical activity, n (%)						
Southeast						
Yes	96 (62.7%)	30 (19.6%)	13 (8.5%)	2 (1.3%)	12 (7.8%)	0.627
No	333 (64.2%)	98 (18.9%)	31 (6.0%)	4 (0.8%)	53 (10.2%)	
South						
Yes	26 (44.8%)	11 (18.9%)	9 (15.5%)	0 (0.0%)	12 (20.7%)	0.066
No	110 (61.1%)	34 (19.0%)	11 (6.1%)	1 (0.6%)	24 (13.3%)	
North and Northeast						
Yes	132 (74.6%)	26 (14.7%)	9 (5.1%)	1 (0.6%)	9 (5.1%)	0.278
No	297 (68.3%)	61 (14.0%)	30 (6.9%)	4 (0.9%)	43 (9.9%)	
Midwest						
Yes	13 (50.0%)	8 (30.8%)	1 (3.8%)	–	4 (15.4%)	0.658
No	61 (60.4%)	21 (20.8%)	6 (5.9%)	–	13 (12.9%)	
Smoking, n (%)						
Southeast						
Yes	51 (50.0%)	25 (24.5%)	8 (7.8%)	2 (2.0%)	16 (15.7%)^a^	0.048
No	429 (66.1%)^c^	119 (18.3%)	41 (6.3%)	4 (0.6%)	56 (8.6%)	
South						
Yes	26 (45.6%)	10 (17.5%)	9 (15.8%)^a^	0 (0.0%)	12 (21.1%)	0.049
No	99 (61.5%)^a^	31 (19.3%)	9 (5.6%)	1 (0.6%)	21 (13.0%)	
North and Northeast						
Yes	34 (68.0%)	5 (10.0%)	4 (8.0%)	1 (2.0%)	6 (12.0%)	0.425
No	297 (70.4%)	62 (14.7%)	26 (6.2%)	3 (0.7%)	34 (8.1%)	
Midwest						
Yes	9 (47.4%)	6 (31.6%)	2 (10.5%)	–	2 (10.5%)	0.405
No	110 (59.8. %)	40 (21.7%)	9 (4.9%)	–	25 (13.6%)	
Hypercholesterolemia, n (%)						
Southeast						
Yes	99 (52.1%)	43 (22.6%)	23 (12.1%)^d^	2 (1.1%)	23 (12.1%)	0.000
No	380 (68.0%)^g^	100 (17.9%)	26 (4.7%)	4 (0.7%)	49 (8.8%)	
South						
Yes	23 (46.9%)	6 (12.2%)	5 (10.2%)	1 (2.0%)	14 (28.6%)^b^	0.009
No	102 (60.4%)	35 (20.7%)	13 (7.7%)	0 (0.0%)	19 (11.2%)	
North and Northeast						
Yes	32 (43.2%)	16 (21.6%)^a^	9 (12.2%)^a^	2 (2.7%)	15 (20.3%)^g^	0.000
No	297 (75.0%)^e^	51 (12.9%)	21 (5.3%)	2 (0.5%)	25 (6.3%)	
Midwest						
Yes	17 (44.7%)	10 (26.3%)	3 (7.9%)	–	8 (21.1%)	0.176
No	102 (61.8%)	36 (21.8%)	8 (4.8%)	–	19 (11.5%)	
Hypertension, n (%)						
Southeast						
Yes	47 (33.6%)	37 (26.4%)^a^	22 (15.7%)^i^	2 (1.4%)	32 (22.9%)^j^	0.000
No	433 (70.9%)^h^	107 (17.5%)	27 (4.4%)	4 (0.7%)	40 (6.5%)	
South						
Yes	19 (33.3%)	11 (19.3%)	7 (12.3%)	1 (1.8%)	19 (33.3%)^f^	0.000
No	106 (65.8%)^f^	30 (18.6%)	11 (6.8%)	0 (0.0%)	14 (8.7%)	
North and Northeast						
Yes	16 (28.1%)	17 (29.8%)^d^	8 (14.0%)	0 (0.0%)	16 (28.1%)^e^	0.000
No	313 (75.8%)^k^	50 (12.1%)	22 (5.3%)	4 (1.0%)	24 (5.8%)	
Midwest						
Yes	9 (26.5%)	10 (29.4%)	4 (11.8%)	–	11 (32.4%)^d^	0.000
No	110 (65.1%)^f^	36 (21.3%)	7 (4.1%)	–	16 (9.5%)	

Data are presented as the means (SD) and N (%)

*DR* diabetic retinopathy, *NPDR* non-proliferative diabetic retinopathy, *BMI* body mass index

* p < 0.006 vs every other DR, ** p < 0.004 vs proliferative DR, *** p < 0.023 vs mild NPDR, ^#^p = 0.032 vs moderate NPDR, ^##^ p < 0.004 vs mild NPDR and proliferative DR, ^###^p < 0.005 vs moderate NPDR, ^+^p<0.003 vs mild and moderate NPDR and proliferative DR. ^++^ p < 0.004 vs mild NPDR

^a^Standard Residual 2.2, ^b^ Standard Residual 3.0, ^c^ Standard Residual 3.1, ^d^ Standard Residual 3.7, ^e^ Standard Residual 5.2, ^f^ Standard Residual 4.2, ^g^ Standard Residual 3.9, ^h^ Standard Residual 8.3, ^i^ Standard Residual 4.9, ^j^ Standard Residual 5.9, ^k^ Standard Residual 7.4

As expected, duration of DM was directly related to severity of DR in all regions of Brazil, p = 0.000. Mean duration of DM was higher in the South and Southeast (17.6 ± 9.9 and 17.0 ± 9.7 years, respectively) and lower in the North and Northeast of Brazil (12.6 ± 7.7 years). On the other hand, levels of HbA1c were greater in the North and Northeast of Brazil (9.3 ± 2.3%) and lower in the Southeast (8.8 ± 2.0%), p = 0.003, and in both regions levels of HbA1c was significantly related to severity of DR, p < 0.012.

Hypertension was higher in the South, p = 0.000, and was related to severity of DR in all regions of Brazil, as was hypercholesterolemia, exception made for the Midwest region. Prevalence of hypercholesterolemia was higher in the Southeast, p = 0.000. Whereas, prevalence of smokers where proportionally higher in the South, p = 0.000.

Regarding the economic status, the Midwest region concentrated the highest proportion of high economic status, the South and Southeast, the medium economic status and the North and Northeast, the low and very low economic status, p = 0.000.

Concerning level of care, in the Southeast the secondary level of care had greater frequency of moderate NPDR, and the tertiary level of care, PDR. In the North and Northeast, the secondary level of care accounted for higher frequency of mild NPDR and PDR, whereas, the tertiary level of care had greater frequency of absence of DR, p = 0.000.

#### Southeast

In the Southeast, patients with moderate NPDR had grater BMI then those with the absence of DR (26.3 ± 4.9 vs 24.0 ± 3.9), p = 0.002. Level of HbA1c was lower in the absence of DR when compared to mild NPDR (8.7 ± 2.0 vs 9.3 ± 2.2%), p < 0.023. Hypercholesterolemia was significantly related to moderate NPDR, p = 0.000 and the proportion of smokers was higher in PDR, p = 0.048.

#### South

In the South, patients with mild DR had higher BMI than those with the absence of DR (26.0 ± 2.8 vs 23.9 ± 4.0), p = 0.004. Hypercholesterolemia was significantly related to PDR, p = 0.009.

#### North and Northeast

In the North and Northeast level of HbA1c was higher in moderate NPDR when compared to absence of DR and mild NPDR (11.1 ± 2.5 vs 9.0 ± 2.1 and 9.1 ± 2.3%, respectively), p < 0.005. Hypercholesterolemia was significantly related to moderate NPDR and PDR, p = 0.000. Level of education was lower in moderate NPDR, 10.0 ± 3.7 years of study, when compared to absent DR, 12.7 ± 3.9 years of study, p < 0.005. More cases of absent DR were identified in the medium economic status, 110 (33.2%), S.Res. 2.8, as was PDR in the low and very low economic status, 29 (72.5%) and 6 (15.0%), respectively, S.Res. 1.8, p = 0.0439.

#### Midwest

In the Midwest region patients with PDR had higher BMI than those with absent DR (25.8 ± 4.5 vs 23.1 ± 4.2), p = 0.010.

### Multinomial regression and stepwise multivariate analysis for NPDR

Multinomial regression and stepwise analyses (Table 4) showed that duration of diabetes and level of HbA1c was significantly related to NPDR in all geographic regions. Hypertension was also statistically significant in the Southeast (2.4, IC 95% of 1.5–3.9, p = 0.000) and North and Northeast (2.4, IC 95% of 1.1–5.3, p = 0.028) of Brazil, whereas BMI was in the South (1.1, IC 95% of 1.0–1.2, p = 0.024). In the Midwest, the odds of NPDR was associated with Caucasian ethnicity (2.8, IC 95% of 1.3–6.1, p = 0.012).

**Table 4:** Final adjusted stepwise analysis with diabetic retinopathy as dependent variable

	Non-proliferative DR				Proliferative DR			
	O.R	CI 95%		p value	O.R	CI 95%		p value
Southeast								
Duration of diabetes, years	1.1	1.0	1.1	0.000	1.1	1.1	1.2	0.000
HbA1c (%)	1.3	1.2	1.4	0.000	1.2	1.0	1.4	0.058
BMI	1.0	1.0	1.1	0.155	1.1	1.0	1.1	0.080
Hypertension	2.4	1.5	3.9	0.000	3.5	1.8	6.6	0.000
Physical activity	0.7	0.5	0.9	0.020	0.6	0.3	1.0	0.040
South								
Duration of diabetes, years	1.1	1.0	1.1	0.002	1.1	1.1	1.2	0.000
HbA1c (%)	1.4	1.1	1.7	0.001	1.1	0.9	1.5	0.318
BMI	1.1	1.0	1.2	0.024	1.0	0.9	1.2	0.980
Hypertension	1.3	0.5	3.2	0.628	5.7	1.9	17.5	0.002
Physical activity	1.7	0.8	3.5	0.154	2.5	0.9	6.7	0.076
North and Northeast								
Duration of diabetes, years	1.1	1.0	1.1	0.001	1.2	1.1	1.3	0.000
HbA1c (%)	1.2	1.1	1.4	0.001	1.2	1.0	1.4	0.041
BMI	1.1	1.0	1.1	0.221	0.9	0.8	1.0	0.063
Hypertension	2.4	1.1	5.3	0.028	5.5	2.1	14.8	0.001
Economic status	0.6	0.4	1.1	0.126	0.2	0.1	0.5	0.002
Midwest								
Duration of diabetes, years	1.1	1.1	1.3	0.000	1.3	1.2	1.4	0.000
HbA1c (%)	1.3	1.1	1.6	0.014	1.3	1.0	1.8	0.084
Caucasian	2.8	1.3	6.1	0.012	0.5	0.2	1.6	0.262

*DR* diabetic retinopathy, *BMI* body mass index

### Multinomial regression and stepwise multivariate analysis for PDR

Considering PDR, multinomial regression and stepwise analyses reveled statistical significance for duration of DM in every geographic region and hypertension in the Southeast, South and North and Northeast. On the other hand, high and medium economic status (0.2, IC 95% of 0.1–0.5, p = 0.002) lowered the risk of proliferative DR in the North and Northeast as did physical activity in the Southeast (0.6, IC 95% of 0.3–1.0, p = 0.043).

### Analysis of variables related to DME by geographic regions

Analysis of variables related to DME by geographic regions of Brazil are shown in Table 5.

**Table 5:** Clinic and laboratory data for diabetic macular edema by geographic region

Variables	Diabetic macular edema		p value
	Yes	No	
Duration of diabetes, years Mean (SD)			
Southeast	19.8 ± 7.5	16.7 ± 9.7	0.047
South	28.7 ± 8.3	17.4 ± 9.9	0.059
North and Northeast	15.0 ± 8.0	12.5 ± 7.7	0.276
Midwest	24.9 ± 10.3	13.3 ± 8.2	0.000
Level of HbA1c (%) Mean (SD)			
Southeast	9.6 ± 2.9	8.8 ± 2.0	0.328
South	10.5 ± 3.3	9.0 ± 2.1	0.059
North and Northeast	12.0 ± 3.1	9.2 ± 2.2	0.009
Midwest	8.8 ± 1.7	9.1 ± 2.1	0.658
BMI Mean (SD)			
Southeast	25.4 ± 4.0	24.5 ± 4.5	0.250
South	25.5 ± 0.8	24.5 ± 3.9	0.409
North and Northeast	23.9 ± 2.8	23.5 ± 3.6	0.718
Midwest	25.1 ± 5.0	23.8 ± 4.4	0.353
Hypertension			
Southeast			
Yes	10 (7.1%)^a^	130 (92.9%)	0.001
No	9 (1.5%)	602 (98.5%)^a^	
South			
Yes	2 (3.5%)	55 (96.5%)	0.168
No	1 (0.6%)	160 (99.4%)	
North and Northeast			
Yes	2 (3.5%)	55 (96.5%)	0.252
No	6 (1.5%)	407 (98.5%)	
Midwest			
Yes	6 (17.6%)^b^	28 (82.4%)	0.016
No	8 (4.7%)	161 (95.3%)^b^	
Hypercholesterolemia			
Southeast			
Yes	10 (5.3%)^b^	180 (94.7%)	0.013
No	9 (1.6%)	550 (75.3%)^b^	
South			
Yes	1 (2.0%)	48 (98.0%)	0.536
No	2 (1.2%)	167 (98.8%)	
North and Northeast			
Yes	3 (4.1%)	71 (95.9%)	0.116
No	5 (1.3%)	391 (98.7%)	
Midwest			
Yes	5 (13.2%)	33 (86.8%)	0.146
No	9 (5.5%)	156 (94.5%)	

Data are presented as the means (SD) and N (%)

*BMI* body mass index

^a^Standard Residual 3.9, ^b^ Standard Residual 2.8

Longer duration of diabetes was significantly related to the presence of DME in the Southeast and Midwest regions (19.8 ± 7.5 vs 16.7 ± 9.7 years, p = 0.047, and 24.9 ± 10.3 vs 13.3 ± 8.2 years, p = 0.000, respectively). Higher level of HbA1c only correlated to the presence of DME in the North and Northeast of Brazil (12.0 ± 3.1 vs 9.2 ± 2.2%, p = 0.009). Still in the North and Northeast, the presence of DME was greater among very low economic status (40.0% vs 7.3%, p = 0.016). In the South, patients who reported physical activity had lower prevalence of DME (5.3% vs 94.7%, p = 0.014). Hypertension was positively related to the presence of DME in the Southeast and Midwest regions (7.1%, p = 0.000 and 17.6%, p = 0.016). Hypercholesterolemia was only significantly related to DME in the Southeast (5.3%, p = 0.013).

After, multinomial regression and stepwise analyses, the variables that reached statistical significance was hypertension in the Southeast (5.6, IC 95% of 2.2–14.4, p = 0.000), HbA1c in the North and Northeast (1.5, IC 95% of 1.2–2.0, p = 0.001) and duration of diabetes in the Midwest (1.1, IC 95% of 1.1–1.2, p = 0.000). Model was not applied in the South because of the limited number of cases of DME.

### Multinomial regression and stepwise multivariate analysis for NPDR, PDR and DME with region as independent variable

When multinomial regression was applied, and region was considered an independent variable, prevalence of NPDR was not statistically different in each geographic region, on the other hand, in the Midwest, prevalence of PDR (2.4, IC 95% of 1.1–5.1, p = 0.022), and DME (7.4, IC 95% of 2.0–27.6, p = 0.003) was significantly higher.

## Discussion

Data on the prevalence of DR will vary according to study methodology, clinic and demographic data of the participants, this fact, combined with the diverse social and cultural reality of the five geographic regions of Brazil, reflects the overall results presented by this study. The North and Northeast at initial analyses had lower prevalence of DR as they had lower mean duration of diabetes. On the other hand, the South had higher prevalence of PDR as they had greater mean duration of DM as well as higher prevalence of hypertension. Adjusting our model to investigate actual differences in the prevalence of DR between the five geographic regions of Brazil, we found no variation in the matter of NPDR. However, the Midwest region concentrated more severe cases of this pathology, as the prevalence of PDR and DME was higher in this part of Brazil independently of every other risk factor, age, sex, ethnicity, level of education, economic status, duration of diabetes, HbA1c, BMI, level of exercise, smoking, hypertension, hypercholesterolemia and level of care. What leads to a more severe DR in patients with T1D in the Midwest region has yet to be enlightened.

As a country with continental territory, racial and cultural miscegenation, Brazil experiences great social economic problems such as social inequality. The human development index (HDI) revels profound differences within the country. The Brazilian Federal District, located at the Midwest region, has the highest HDI, 0.839, considered a very high index. On the other hand, the North and Northeast has half of its counties leveled at a medium HDI, with index as low as 0.667 in 2014, not to mention level of education, reflecting levels of low HDI in two of its counties [15]. Other than economic and social development differences, cultural habits that differ in each of the five regions of Brazil may influence risk factors for chronic diseases, and therefore severity of DR, related to eating habits and lifestyle [16]. This combination of realities generates a complex and multifactorial panorama, that challenges health approach in Brazil.

The prevalence of any DR and PDR in each geographic region of Brazil correlates with the prevalence estimated from a pooled meta-analysis in patients with T1D [17], which varied from 20.53 to 0.37%, respectively, in patients with less than 10 years duration of DM to 55.55 and 19.46%, respectively, in patients with DM duration between 10 and less than 20 years. In the meta-analysis above mentioned, prevalence of DME in patients with T1D and duration of diabetes between 10 and less than 20 years was 12.27%. In our study, prevalence of DME in the Midwest region of Brazil more closely correlates to the global estimates, but prevalence of maculopathy in the South, Southeast and North and Northeast was lower than expected.

As presumed and vastly proven [18, 19], time of diabetes in our study was directly related to the presence and severity of DR in all studied regions and to DME in the Southeast and Midwest. These results reinforce the role of duration of diabetes as a major risk factor in the development and severity of DR.

In every geographic region, the glycemic control was an independent variable in the development of DR in our study, fact proven since the DCCT and by several other studies, highlighting the HbA1c as a major modifiable risk factor [20–23]. Non of the studied regions achieved mean HbA1c within the recommended level for adequate clinical and metabolic control established by the Brazilian Diabetes Society and ADA [9, 24] and only the Southeast achieved mean levels lower to 9.0%, that defines poor glycemic control. We emphasize the special attention the glycemic control deserves in the North and Northeast of Brazil, since patients presented higher mean levels of HbA1c and glycemic control was an independent risk factor for DME.

A positive relation between DR and hypertension has been constantly demonstrated [25–27]. Prevalence of hypertension was higher in the South, where the risk of PDR was 5 times higher in the presence of hypertension as was the risk of DME in the Southeast. As a well-known major modifiable risk factor in the development and severity of DR, hypertension proved to play an important role in DR in the South, Southeast and North and Northeast of Brazil. Hypercholesterolemia was related to the presence and severity of DR in the South, Southeast and North and Northeast but had no strength as an independent factor.

BMI is still not clearly linked to the presence and severity of DR in patients with T1D. In our study, overweight was noticed among those with NPDR in the Southeast and PDR in the Midwest region at a significant level and should be a factor to be observed. The roll of smoking is also controversy, but in our study the South and Southeast ought to be alert as smoking was a contributing factor to DR [28].

Our study shows the diverse economic reality of each geographic region and best correlates it with DR in the Northeast, where the high and medium economic status had 80% less chance of PDR and the prevalence of DME was higher in the very low economic status. Data from a National Survey in the five geographic regions of Brazil showed that both the quality of health as the use of health services in the North and Northeast of Brazil were at significant lower levels when compared to the other geographic regions, South, Southeast and Midwest [29]. Substandard access to health care in this part of Brazil is a well known reality and will reflect in every aspect of the individual’s health combined with social inequality, where individuals at social vulnerability will have less access to the health care system, public or private.

In Brazil, the public health system is structured in three levels of care and patients should move from the first towards the third level of care according to disease complexity. In the North and Northeast, the second level of health care concentrated higher frequency of PDR than the third level, which, in the other hand, concentrated higher frequency of absence of DR, an inversion of flow of how the public health system was designed to work in Brazil, where the tertiary level should account for the most severe cases and where the system is structured to deal with the complex treatment it will demand. As expected, our study revels signs of a public health care system better organized in the Southeast, where the tertiary level of care accounted for more cases of PDR.

At last, independently of DR, the studied population presented other ophthalmological pathologies, such as cataract and glaucoma, what leads us to reinforce the essential role of regular ophthalmological examination as a routine in the health care of patients with DM.

To our knowledge, this is the first large sample size study that aims to study the prevalence of DR and its risk factors in each of the five geographic regions of Brazil. With only patients with T1D, represents the diverse young T1D population of Brazil, with a wide range of ethnic groups and economic backgrounds from all geographic regions of the country. This constitutes the primary strength of this study.

Our study has some limitations that must be mentioned. The first one would be the classification of DME without slit lamp examination, which could have underestimated the prevalence of maculopathy in our results. Since retinopathy classification was not stated by the same examiner it could be listed as a limitation although they were all performed by retina specialist with combined training in the same university center. The lower number of participants at the North of Brazil led us to combine data with the Northeast, this fact limits our analysis to distinguish prevalence of DR in these two important geographic region of Brazil. The diagnoses of DM at clinical bases could also be stated as a limitation.

## Conclusions

We conclude that the prevalence of NPDR in patients with T1D was no different between each geographic region of Brazil. Furthermore, a higher prevalence of PDR and DME, which are considered as sight threatening stages of the disease, was noticed in the Midwest region and deserves further investigations by other studies. Duration of DM is of central importance to all, as is glycemic control, that should receive better effort to have its level lowered by every geographic region, particularly at the North and Northeast. Hypertension is another fundamental factor to every region, at special in the South and Southeast. Finally, patients in social and economic vulnerability deserves special attention in the North and Northeast of Brazil.

## Abbreviations

DR: diabetic retinopathy
T1D: type 1 diabetes
PDR: proliferative diabetic retinopathy
DME: diabetic macular edema
ADA: American Diabetes Association
SUS: Sistema Único de Saúde—Brazilian National Health Care System
BMI: body mass index
NPDR: non proliferative diabetic retinopathy
IBGE: Brazilian Institute of Geography and Statistic Census
S.Res.: Standard Residual
HDI: human development index
